# Exploring the Ability of Luminescent Metal Assemblies to Bind and Sense Anionic or Ionizable Analytes A Ru(phen)_2_bipy-Based Dizinc Complex for Bisphenol A (BPA) Recognition

**DOI:** 10.3390/molecules26030527

**Published:** 2021-01-20

**Authors:** Luca Conti, Liviana Mummolo, Giammarco Maria Romano, Claudia Giorgi, Gina Elena Giacomazzo, Luca Prodi, Andrea Bencini

**Affiliations:** 1 Department of Chemistry “Ugo Schiff”, Università degli Studi di Firenze, via della Lastruccia 3, 50019 Sesto Fiorentino, Italy; luca.conti@unifi.it (L.C.); giammarcomaria.romano@unifi.it (G.M.R.); ginaelena.giacomazzo@unifi.it (G.E.G.); 2 Department of Chemistry “Giacomo Ciamician”, Università degli Studi di Bologna, via Selmi 2, 40126 Bologna, Italy; liviana.mummolo2@unibo.it

**Keywords:** bisphenol A, ruthenium complexes, anion binding, supramolecular chemistry, zinc complexes, luminescent chemical sensors, mixed metal complexes

## Abstract

The synthesis of a new Ru^II^ complex, in which the metal is coordinated by two 1,10-phenanthroline ligands and a 2,2′-bipyridyl unit linked, via methylene bridges in its 4 and 4′ positions, to two 1,4,7,10-tetraazacyclododecane (cyclen) macrocycles ([Ru(phen)_2_**L**]^2+^) is reported. Protonation and Zn^II^ binding by [Ru(phen)_2_**L**]^2+^ have been analyzed by potentiometric titration, evidencing the formation of mixed hetero-binuclear and hetero-trinuclear Zn^II^/Ru^II^ complexes. These complexes were tested as bis-phenol A (BPA) binders. Only the dizinc complex with [Ru(phen)_2_**L**]^2+^ is able to bind BPA in aqueous solution, affording a remarkably stable {Zn_2_[Ru(phen)_2_**L**]BPA(H_−2_)}^4+^ adduct at neutral pH, in which BPA is bound in its doubly deprotonated form to the two Zn^II^ ions. BPA binding was found to quench the luminescence emission of the Ru^II^(phen)_2_bipy core. Although the quenching effect is modest, this study demonstrates that appropriately designed dizinc complexes can be used for binding and optical sensing of BPA in water.

## 1. Introduction

Bisphenol A (BPA) is used in large amounts in the production of polycarbonates, epoxy resins and thermal paper [1,2,3] and, therefore, it is commonly contained in various products for everyday use, such as food contact materials, including packaging, bottles and lacquers coatings for tins, electronic equipment, paper or toys [4,5,6]. On the other hand, it has been now recognized that BPA is able to damage endocrine functions through mimicking or blocking endogenous hormones [7,8,9,10,11,12]. As for many Endocrine Disrupting Chemicals (EDCs), BPA has been intensively introduced into the environment in the last decades, leading to the exposure of animals and humans to its toxic action. In this panorama, in the last few years several approaches have been used to develop efficient tools to bind and/or detect BPA, including Au or silica nanoparticles coupled with aptameric binding sites [12,13,14,15,16], electrochemical biosensors [17,18], and molecular imprinted nanoparticles or polymers [19,20,21]. Less attention has been devoted to synthetic molecular receptors for BPA recognition and sensing by using a supramolecular approach, in which an appropriately designed molecular guest can bind BPA to afford a host–guest complex stabilized by multiple weak interactions. In this context, in fact, the only examples reported in the literature are represented by *β*-cyclodextrin-containing materials for BPA separation [22,23,24,25,26]. In host–guest adducts, eventual changes of a physical property of the receptor occurring upon complex formation can be exploited for BPA detection. From this point of view, BPA contains a couple of phenolic -OH functions, which, in principle, can give interactions with hydrogen bonding acceptor sites of a receptor. Hydrophobic and/or π-stacking interactions, involving the aromatic portions of BPA, can also be exploited to stabilize the complex. On the other hand, these interactions can be too weak to afford stable host–guest adducts [27,28], especially in water solutions. For instance, solvation, in particular in water media, of the OH groups of BPA and of the H-bonding acceptor sites of the receptor normally strongly inhibits the formation of stable hydrogen bonding interactions. A possible different strategy is represented by the employment of metal complexes in which the metal ion acts as anchoring point for the targeted substrate. BPA is a weak acid [29] and undergoes deprotonation above pH 8 to give mono- and dicharged anions in aqueous solution. It has been shown that easily ionizable analytes, as BPA is, can be conveniently bound by metal complexes in which the coordinated metal possesses free binding sites in its coordination environment [27,28,30,31,32,33]. Coordination of weakly acidic groups, including phenolic OH functions, decreases their pK_a_ values, favouring their deprotonation. Therefore, deprotonation of phenol groups, which normally occurs at alkaline pH values, could take place at lower pH values, affording phenolate anions bound to the metal.

On the basis of these considerations, we designed the new receptor [Ru(phen)_2_**L**]^2+^, which contains two 1,4,7,10-tetraazacyclododecane (cyclen) units linked by a Ru^II^(phen)_2_(bipy) core (phen = 1,10-phenanthroline, bipy = 2,2′-bipyridyl). The idea on which we based the design of our system is that cyclen could form stable complexes with first-row transition metals, which are tetracoordinated by the four amine groups of the macrocycle [34], while the luminescent Ru^II^(phen)_2_(bipy) moiety [35] could be exploited as a signalling unit. In particular, the presence of two cyclen units could lead to the formation of binuclear complexes with transition metals, in which each coordinated cation can be used as binding site for a single phenolate function of BPA in an overall bridging coordination mode. This would afford stable ternary complexes, which could potentially affect the emission properties of the Ru^II^(phen)_2_(bipy) unit.

Among transition metals, Zn^II^ appears particularly promising as a binding site for exogenous species. In fact, this metal cation can easily expand and change its coordination sphere, achieving coordination number greater than four. Furthermore, Zn^II^ is not paramagnetic and, therefore, it normally does not quench the emission of luminophores. This approach has been used by Kimura and co-workers to develop a tris-bipyridyl Ru^II^ complexes, containing three bipy units each functionalized with two cyclen units, whose hexa-nuclear Zn^II^ was used for the recognition of triphosphate nucleosides [36].

**Scheme 1:**
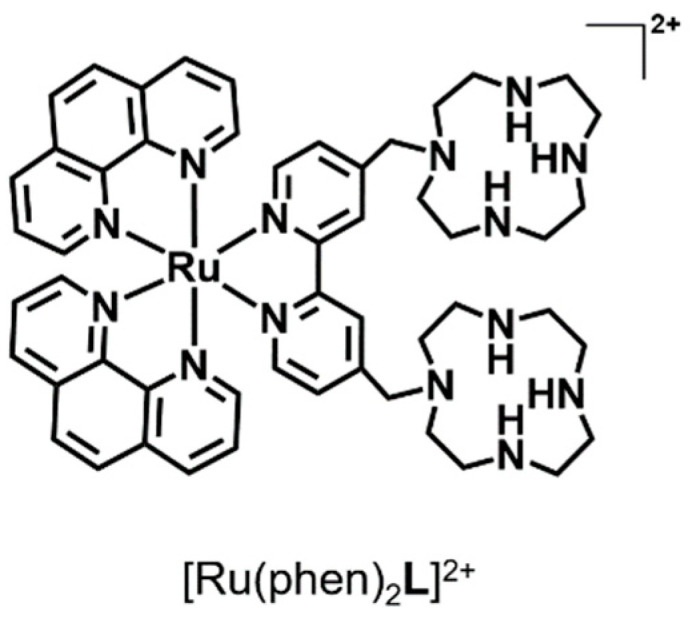
Sketch of the [Ru(phen)_2_**L**]^2+^ complex.

In this paper, we have synthesized the new complex [Ru(phen)_2_**L**]^2+^ (Scheme 1), which contains a bipyridyl unit with two appended cyclen macrocycles (herein indicated with **L**). The ability of its Zn^II^ complexes to form ternary complex with BPA was analysed as well as the emission properties of the dizinc complex in the presence of this substrate.

## 2. Results and Discussion

### 2.1. Synthesis of the Ru^II^ Complex

**Figure 1:**
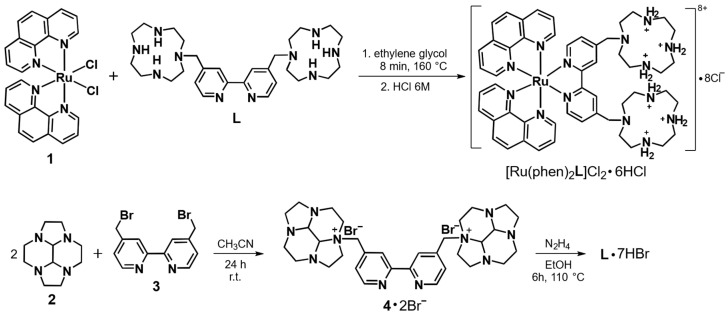
Synthetic pathway to obtain [Ru(phen)_2_**L**]^2+^.

The ruthenium complex [Ru(phen)_2_**L**]^2+^ was obtained by using complex **1** as synthetic precursor. Reaction of **1** with the bipyridyl-based ligand **L** in ethylene glycol at 180 °C leads to the substitution of the two chloride anions with the heteroaromatic nitrogens of **L** affording, after treatment with 6 M HCl, [Ru(phen)_2_**L**]^2+^ as hexa-protonated octa-chloride salt (Figure 1) [37]. More interestingly, ligand **L** was obtained by using the bisaminal procedure, which provides, among others, an easy tool to produce systems composed by two linked polyamine moieties, including two cyclen macrocycles [38]. In fact, the bisaminal conformation confers, to the couple of tertiary amines located in opposite positions, higher reactivity toward electrophyles. At the same time, reaction of the first tertiary amine group leads to the formation of a positively charged monocation, which is less prone to react with a second electrophilic agent. As a matter of fact, one of four amine group is more reactive toward electrophilic agents. This can be exploited to link two cyclen moieties with a chosen bridging group. Reaction of the bisaminal derivative decahydro-2a,4a,6a,8a-tetraaza-cyclopenta[fg]acenaphthylene **2** with 4,4′-bis(bromomethyl)- 2,2′-bipyridyl 3 in 2:1 molar ratio in CH_3_CN affords as unique product the dibromide salt of the ammonium cation 4, in good yields (>70%) (Figure 1). This procedure also takes advantage of the extremely low solubility of the salt, which can be isolated by simple filtration from the reaction mixture. Compound **4** was practically pure and was used in the following reaction without further purification. Its deprotection with hydrazine affords the bis-macrocycle **L**, which was finally purified by treatment with HBr 48% in EtOH and isolated as hepta-hydrobromide salt.

### 2.2. Speciation Study

To verify the effective ability of the Zn^II^ complexes with [Ru(phen)_2_**L**]^2+^ to bind BPA, we performed first a speciation study, by means of potentiometric titrations, on the complexes formed by [Ru(phen)_2_**L**]^2+^ with Zn^II^, with the final purpose of determining the eventual ternary complexes formed with BPA and their stability constants in aqueous solution.

**Figure 2:**
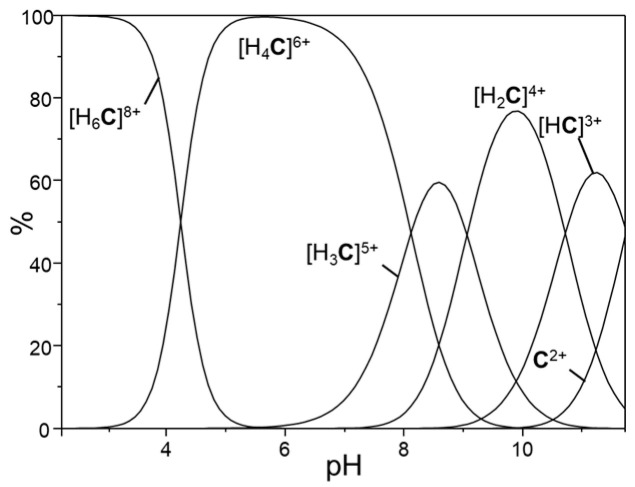
Distribution diagram of the protonated species of complex [Ru(phen)_2_**L**]^2+^ (the [Ru(phen)_2_**L**] unit is indicated with **C** for clarity).

As the process of metal coordination in water competes with that of ligand protonation, we necessarily determined the protonation constants of [Ru(phen)_2_**L**]^2+^. Their values are given in Table 1, while Figure 2 reports the distribution diagrams of the protonated forms of **L** at different pH values. The pK_a_ values of BPA, determined in our experimental conditions, are also shown in Table 1.

The basicity properties of [Ru(phen)_2_**L**]^2+^ reflect the presence of two cyclen moieties within its framework. In fact, its first two protonation constants are somewhat lower than the first one of cyclen (log K = 11.27) [39] and rather similar to each other, as expected considering that the two H^+^ ions in [Ru(phen)_2_(H_2_**L**)]^4+^ are likely to be located on two different macrocyclic moieties. The lower values observed with respect to cyclen are due to the presence in [Ru(phen)_2_**L**]^2+^ of a positively charged metal cation.

Analogously, [Ru(phen)_2_**L**]^2+^ displays third and fourth protonation constants, similar to each other, which are somewhat lower than the second basicity constant of cyclen, (log K = 9.8). In the [Ru(phen)_2_(H_4_**L**)]^6+^ cation, the most abundant species at neutral pH values (Figure 2), each macrocyclic unit is likely to be in its diprotonated form. Finally, cyclen, shows a poor tendency to bind a third H^+^ ion. Once again, **L** parallels the behaviour of this macrocycle. In fact, binding of two further H^+^ ions occurs only at markedly acidic pH values (Figure 2). In this case, however, only the constants for the simultaneous addition of two H^+^ ions to the [Ru(phen)_2_(H_4_**L**)]^6+^ can be determined, as generally observed when two protonation equilibria take place in very similar pH regions. As far as the BPA acidity is concerned, the pK_a_s determined in our experimental condition are in good agreement with those reported in the literature [29].

**Figure 3:**
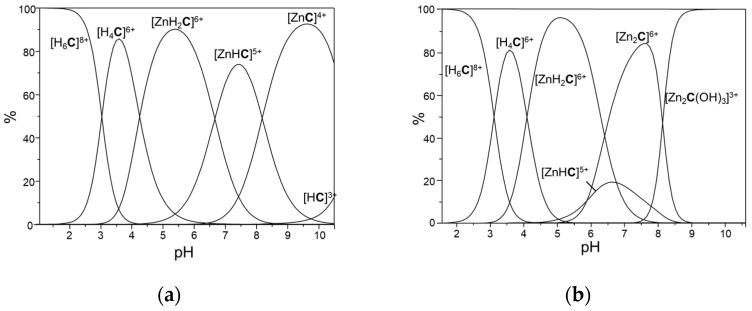
Distribution diagrams of the complexes formed by the [Ru(phen)_2_**L**]^2+^ (the [Ru(phen)_2_**L**] unit is indicated with **C** for clarity); (**a**) [Ru(phen)_2_**L**]^2+^/Zn^II^ 1:1 molar ratio; (**b**) [Ru(phen)_2_**L**]^2+^/Zn^II^ 1:2 molar ratio ([Ru(phen)_2_**L**]^2+^ = 1·10^−3^ M).

As previously anticipated, cyclen affords stable complexes with Zn^II^ [34,40,41], also avoiding the formation of not complexed Zn-OH species. Consequently, [Ru(phen)_2_**L**]^2+^ forms stable mono- and binuclear complexes in aqueous solution, as shown in Table 2 and Figure 3. The Ru^II^ complex gives a mononuclear Zn^II^ complex, {Zn[Ru(phen)_2_**L**]}^4+^ somewhat less stable than the corresponding complex with cyclen (log K = 14.75 vs. 16.2 [42] for the formation of {Zn[Ru(phen)_2_**L**]}^4+^ and [Zn(cyclen)]^2+^, respectively), as expected considering both the electrostatic repulsion between the two metals in {Zn[Ru(phen)_2_**L**]}^4+^ and the presence of a tertiary amine group within the cyclen unit, which normally displays a lower binding ability than a secondary one. However, the formation of Zn^II^-OH complexes was not detected by potentiometric measurements, nor was Zn(OH)_2_ precipitation observed. At the same time, {Zn[Ru(phen)_2_**L**]}^4+^ shows a marked tendency to bind up to two H^+^ ions. The corresponding protonation constants are higher than 7 log units and reflect the presence of a cyclen unit not bound to the metal, which can be used either to bind acidic protons or a second Zn^II^ ion. As a result, in the presence of 1 equiv. of Zn^II^, the protonated species {Zn[Ru(phen)_2_(H**L**)]}^5+^ and {Zn[Ru(phen)_2_(H_2_**L**)]}^6+^ are the most abundant complexed species formed in solution from acidic to alkaline pH values (Figure 3a), while in the presence of two equivs. of Zn^II^, dinuclear species are formed from neutral to alkaline pH values (Figure 3b).

The {Zn_2_[Ru(phen)_2_**L**]}^6+^ complex, formed at neutral pH values, shows a marked tendency to give hydroxylated species, due to deprotonation of water molecules bound to the Zn^II^ ion, as often found for Zn^II^ complexes in which the metal presents a coordination sphere not saturated by ligand donors. The formation of a stable {Zn_2_[Ru(phen)_2_**L**](OH)_3_}^3+^ is actually observed at slightly alkaline pH values. The three deprotonation processes, which originate the final three-hydroxo complex, are not singularly detected, probably due to their formation in solution at very similar pH values. 

**Figure 4:**
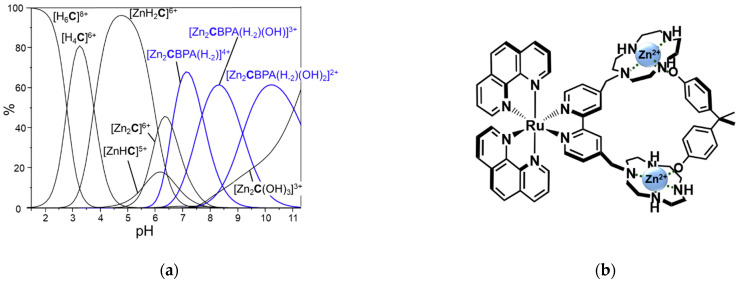
(**a**) Distribution diagrams of the complexes formed by the [Ru(phen)_2_**L**]^2+^ (the [Ru(phen)_2_**L**] group is indicated with **C** for clarity) with Zn^II^ and BPA 1:2:1 molar ratio ([Ru(phen)_2_**L**]^2+^ = 1.10^−3^ M); (**b**) proposed structure of the BPA adduct with the dizinc complex.

The dizinc complex contains two well separated metal ions, both containing free binding sites for substrate coordination and, therefore, appears to be an appropriate receptor for BPA. Indeed, potentiometric titrations show that {Zn_2_[Ru(phen)_2_**L**]}^6+^ can bind, at neutral pH (Figure 4a), BPA in its dianionic form, BPA(H_−2_)^2−^. In the alkaline pH region, the formation of hydroxylated ternary complexes, still containing the BPA(H_−2_)^2−^, is also observed. Interestingly enough, potentiometric measurements performed in the absence of Zn^II^ point out that the Ru-based ligand does not give detectable interactions with BPA at any pH values. Similarly, the mononuclear Zn^II^ complexes do not coordinate BPA. Hydrogen bonding between the phenolic moieties of BPA and the ammine/ammonium groups of the receptor or coordination of BPA to a single metal cation are not sufficient to provide the formation of host–guest adducts in aqueous solution. Instead, the presence of two spaced metal cations, able to simultaneously interact with the two phenolic functions of the substrate, is the necessary requirement to obtain double deprotonation of BPA and its coordination to the dizinc receptor. This strongly suggests that BPA is bound by the {Zn_2_[Ru(phen)_2_**L**]}^6+^ complex in a bridging coordination mode, as sketched in Figure 4b.

The formation of the BPA adduct {Zn_2_[Ru(phen)_2_L]BPA(H_−2_)}^4+^ is also confirmed by an ESI mass spectrum recorded on an aqueous solution at pH 7 containing the dizinc complex with the Ru^II^-based ligand and BPA in 1:1 molar ratio (Figures S11–S15). The spectrum shows a peak at 335.6003 da, attributable to the tetracharged {Zn_2_[Ru(phen)_2_L]BPA(H_−2_)}^4+^ (*m*/*z* = 4). Although this peak is formed in a “complex” spectral region, containing peaks derived from fragmentation of the ligand or of its dizinc complex, it can be safely attributed to the complex with BPA, thanks to the good agreement between the predicted and experimentally isotopic pattern. Similarly, a peak at 446.4632 Da (*m*/*z* = 3) can again be attributed to the complex with BPA.

### 2.3. Photophysical Properties

**Figure 5:**
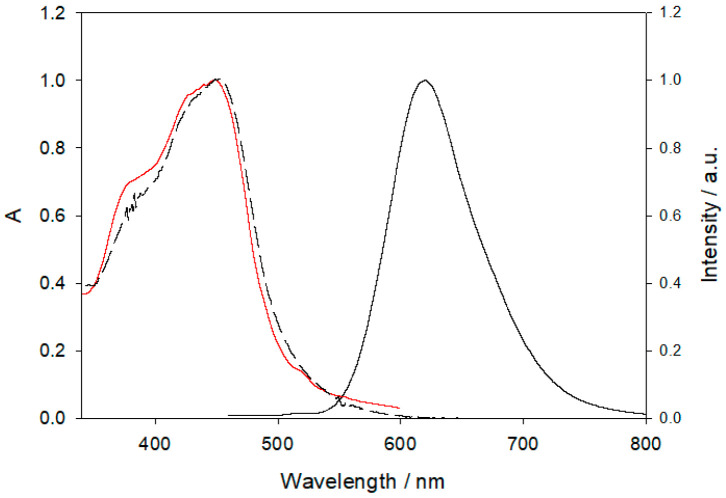
Normalised absorption (black dashed line), emission (black line) and excitation (red line) spectra of [Ru(phen)_2_**L**]^2+^.

[Ru(phen)_2_**L**]^2+^ shows, in water solution, the characteristic absorption and luminescence properties expected for polypyridine Ru complexes (Figure 5).

**Figure 6:**
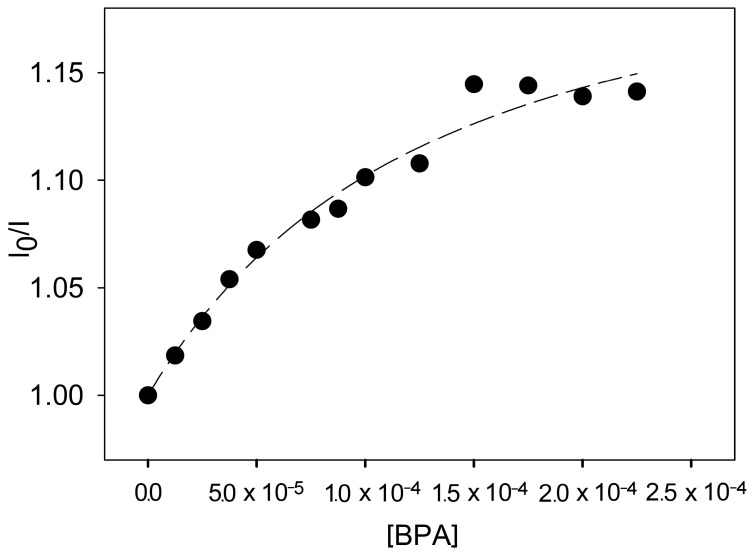
Ratio of luminescence intensity of the [Ru(phen)_2_**L**]^2+^ complex over luminescence intensity of the complex upon addition of increasing amounts of BPA at pH = 7.4 (black dots, λ_exc_ = 440 nm; λ_em_ = 620 nm) and fitting curve obtained using equation 1 (black line). ([Ru(phen)_2_**L**]^2+^ = 20 μM, [Ru(phen)_2_**L**]^2+^/[Zn^II^] = 1:2 stoichiometric ratio).

In particular, [Ru(phen)_2_**L**]^2+^ presents an absorption band in the visible region with maximum at 450 nm (e = 11,100 M^−1^ cm^−1^) and an emission band in aerated solution with maximum at 615 nm, a luminescence quantum yield of 0.027, and an excited state lifetime of 460 ns; as expected, the corrected excitation spectrum is proportional to the absorption one in all the spectral range (220–580 nm). It is noteworthy that all the photophysical properties are almost constant in a wide pH range (3–12). Taken together, all these results clearly indicate that the amines present in the two cyclen units are not able to quench the luminescence of the Ru complex, in agreement with what observed, for example, in Ru complexes with the 4-dimethylamino-2,2′bipyridine ligand [43]. In this context, the addition of two equivalents of Zn^II^ ion at pH 7.4 does not cause noticeable intensity changes, indicating a negligible electronic effect on the bipy ligand caused by complexation that leads to the formation of {Zn_2_[Ru(phen)_2_**L**]}^6+^. On the contrary, the addition of an increasing amount of BPA to the system containing [Ru(phen)_2_**L**]^2+^ at a concentration of 20 μM with Zn^II^ in a 1:2 stoichiometric ratio caused a quenching of the luminescence intensity of the Ru complex, as it can be seen in Figure 6. This intensity decrease is not accompanied by a concomitant change in the excited state lifetime, ruling out the contribution from a dynamic quenching mechanism, and in agreement with the formation of a stable adduct indicated by the speciation study discussed above.

Comparison can be made with the system designed by Kimura and co-workers, containing a Ru^II^ centre coordinated to three bipyridyl units, each linked, via methylene bridges in the 3 and 3′ positions, with two cyclen units [36]. Similarly to the present case, each macrocyclic moiety firmly binds Zn^II^ at neutral pH, while hydroxo Zn^II^-OH species are formed at alkaline pH values. The resulting hexa-nuclear Zn^II^ complex was able to bind compounds inositol 1,4,5-triphosphates (ITP) and *cis,cis*-1,3,5-cyclohexanetriol triphosphate (CTP_3_) to form 1:1 and 1:2 adducts. Differently from the present case, both anions use their phosphate groups to bridge three Zn^II^ ions bound to cyclen macrocycles linked to 3 different bipyridyl moieties and the binding event leads to emission enhancement.

From an analytical point of view, the observed intensity changes suffer from a limited overall quenching even when BPA is in an equimolar amount respect to [Ru(phen)_2_**L**]^2+^; despite this limitation, it is however possible to detect the presence of BPA in the low μM range. The results obtained so far, however, demonstrated that this strategy can be promising for the detection of BPA even at lower concentrations, and the data reported here can be used for an improved design of the sensing structure.

## 3. Conclusions

Appropriately designed dizinc complex can be exploited to develop new molecular receptors and signalling systems for BPA in aqueous solution. While [Ru(phen)_2_**L**]^2+^ and its mononuclear Zn^II^ adduct are totally unable to bind BPA, the dizinc complex gives a stable 1:1 adduct in aqueous solution at neutral pH, in which the BPA, in its bis-anionic form, is bound to the cyclen-coordinated Zn^II^ ion. Therefore, the presence of two adequately spaced Zn^II^ ions favours deprotonation of both phenolic function and their simultaneous binding to the metals in a bridging coordination fashion. The Ru^II^(phen)_2_bipy core can act not only as a spacer unit but also as a signalling moiety. Although the observed change in luminescence emission upon BPA binding is not exceptional, this study demonstrates that the use of simple metal complexes based on a Ru^II^ core represents a promising approach to develop more efficient luminescent reporters for elusive analytes in aqueous solution, such as BPA.

## 4. Materials and Methods

### 4.1. General Procedures

Compounds decahydro-2a,4a,6a,8a-tetraaza-cyclopenta[*fg*]acenaphthylene (**2**) [37], 4,4′-bis(bromomethyl)-2,2′-bipyridine (**3**) [44] were prepared in accordance with the methods described in the literature. NMR spectra were recorded on a Bruker 400 MHz (Bruker Corp.: Billerica, MA, USA) instrument. ESI-mass spectra were collected on a TSQ 700 Finnigan Mat (Finnigan-MAT Corp.: San Jose, CA, USA) or ABSciex triple TOF 5600+ (AB Sciex: Framingham, MA, USA) equipments. Reagents and solvents were from Sigma-Aldrich: Oakville, ON, USA).

### 4.2. Synthesis of Bis[decahydro-4a,6a,8a-Triaza-2a-Azoniacyclopent[fg]acenaphthylene],2a,2′a-[2,2′-bipyridine-4,4′-Diylbis(methylene)] (***4***·2Br^−^)

A mixture of the bisaminal derivative decahydro-2a,4a,6a,8a-tetraaza-cyclopenta[*fg*]acenaphthylene (**2**) (540 mg, 2.78 mmol) and 4,4′-bis(bromomethyl)- 2,2′-bipyridine (**3**) (480 mg, 1.40 mmol), in 15 mL of dry CH_3_CN, was stirred at room temperature for 24 h under a nitrogen atmosphere. The solid product **4**·2Br^−^ formed as a white solid was collected by filtration, washed with CH_3_CN and dried in vacuum. The di-bromide salt of compound **4**·2Br^−^ was used in the following synthetic step without further purification. Yield 736 mg (72%).

Anal. calcd. for C_32_H_46_Br_2_N_10_: C 52.61, N 19.17, H 6.35; found C 52.53, N 19.08, H 6.46.^1^H-NMR (CD_3_OD, 400 MHz, Bruker ARX-400): δ(ppm) 8.92 (d, 2H, *J*_6-5_ = 4.8 Hz): H_6,6′_ (bpy); 8.74 (s, 2H): H_3,3′_ (bpy); 7.81 (d, 2H, *J*_5-6_ = 4.8 Hz): H_5,5′_ (bpy); 5.20 (d, 2H, *J* = 13.2 Hz): H_8b-8′b_ or H_8c-8′c_ (glyoxal); 4.96 (d, 2H, *J* = 13.2 Hz); H_8b-8′b_ or H_8c-8′c_ (glyoxal); 4.43–4.37 (m, 2H); 4.16 (s, 2H); 3.89–3.84 (m, 4H): -CH_2_ (methylen bridge); 3.71–3.65 (m, 2H); 3.55–3.52 (m, 4H); 3.42–3.37 (m, 2H); 3.32–3.29 (m, 4H); 3.21–3.18 (m, 2H); 3.06–2.89 (m, 8H); 2.80–2.77 (m, 2H); 2.66–2.56 (m, 4H) (Supplementary Material, Figure S1).^13^C-NMR (CD_3_OD, 400 MHz, Bruker ARX-400) δ(ppm) 157.60; 151.93; 139.30; 128.84; 125.71; 85.45; 72.93; 62.92; 60.43; 60.35; 59.15; 52.88; 44.46 (Supplementary Material, Figure S2).

### 4.3. Synthesis of 4,4′-Bis[methylen-(1,4,7,10-Tetraazacyclododecane)]-2,2′-Bbipyridine (***L***)

Compound **4**·2Br^−^ (700 mg, 0.96 mmol) was dissolved in 10 mL of hydrazine and 2 mL of ethanol and the mixture was stirred at 110 °C for 6 h. After cooling at room temperature, the solvent was vacuum evaporated under reduced pressure to afford a yellow solid compound, which was dissolved in NaOH 15 M aqueous solution (10 mL). The resulting solution was extracted with chloroform (4 × 20 mL) and the organic layers were collected and dried over Na_2_SO_4_. After solvent removal under vacuum a colorless oil was obtained. The product was dissolved in ethanol (10 mL) and HBr (48%, 1 mL) was dropwise added to the resulting solution, affording the hepta-hydrobromide salt of **L** (**L**·7HBr·2H_2_O) as a yellow solid. Yield 812 mg (75%).

Anal. calcd. for C_28_H_59_Br_7_N_10_O_2_: C 29.84, N 12.43, H 5.28; found C 29.78, N 12.38, H 5.31; ^1^H-NMR (D_2_O + DCl, pD < 2, 400 MHz, Bruker ARX-400): δ(ppm) δ 8.87 (d, 2H, *J*_6-5_ = 5.5 Hz): H_6,6′_ (bpy); 8.51 (s, 2H): H_3,3′_ (bpy); 7.94 (d, 2H, *J*_5-6_ = 5.4 Hz): H_5,5′_ (bpy); 4.17 (s, 4H): -CH_2_ (methylen bridge); 3.36–3.32 (m, 8H); 3.24–3.20 (m, 8H); 3.05–2.99 (m, 16H) (Supplementary Material, Figure S3). ^13^C-NMR (D_2_O + DCl, pD < 2, 400 MHz, Bruker ARX-400): δ(ppm) 153.02; 148.47; 146.93; 128.45; 125.82; 56.40; 48.31; 45.21; 42.66; 42.19 (Supplementary Material, Figure S4).

### 4.4. Synthesis of [Ru(phen)_2_***L***]Cl_2_·6HCl

A quantity of 260 mg of **L**·7HBr (0.24 mmol) was passed through an anion-exchange resin (Dowex 1 × 4 − 50 mesh-chloride form, Sigma Aldrich: Oakville, ON, USA) to obtain the macrocycle in its “HBr-free”-not protonated amine form (**L**). Then, equimolar amounts of Ru(phen)_2_Cl_2_ (**1**) (117.2 mg, 0.22 mmol) and of the polyazamacrocyle **L** (115.4 mg, 0.22 mmol) were suspended in ethylene glycol (7 mL) and heated in a microwave reactor (60 W) at 160 °C for 8 min. The dark-violet suspension became a clear deep orange solution indicating the formation of the ruthenium complex. The mixture was then cooled to room temperature and the solvent was removed by distillation under reduced pressure. The resulting red-orange solid residue was dissolved in a minimal amount of HCl 6 M (2 mL). Addition of EtOH led to precipitation of a red-orange solid which was collected by filtration and washed with acetone/ethyl ether. Double recrystallization from acid HCl-water/acetone made it possible to obtain the final product as hexa-protonated octa-chloride salt [Ru(phen)_2_**L**]Cl_2_·6HCl·2H_2_O. Yield 180 mg (59%).

Anal. calcd. for C_52_H_74_Cl_8_N_14_O_2_Ru: C 47.61, N 14.95, H 5.69; found C 47.59, N 14.98, H 5.71; ^1^H-NMR (D_2_O + DCl, pD < 2, 400 MHz, Bruker ARX-400): δ(ppm) 8.67 (d, 2H, *J* = 8.28 Hz); 8.61–8.53 (m, 4H); 8.33–8.20 (m, 6H); 7.93 (d, 2H, *J* = 4.48 Hz); 7.84–7.75 (m, 4H); 7.58–7.52 (m, 2H); 7.29 (d, 2H, *J* = 4.52 Hz); 4.52 (s, 4H, -CH_2_); 3.37–3.19 (m, 8H); 3.19–3.09 (m, 8H); 3.04–2.94 (m, 8H); 2.94–2.76 (m, 8H) (Supplementary Material, Figure S5). ^13^C-NMR (D_2_O + DCl, pD < 2, 400 MHz, Bruker ARX-400) δ(ppm) 158.26; 153.20; 152.70; 148.44; 148.21; 146.79; 141.26; 137.67; 137.53; 137.44; 131.54; 128.62; 126.46; 126.17; 125.52; 56.00; 53.04; 51.03; 49.89; 49.74; 49.56; 48.22; 48.09; 47.29; 46.80; 45.14; 44.52; 43.73; 43.42; 42.60; 42.16; 41.68 (Supplementary Material, Figure S6). ESI Mass spectrum: 493.236 (*z* = 2, [Ru(phen)_2_**L**]^2+^); 513.743 (*z* = 2, [Ru(phen)_2_**L** + CH_3_CN]^2+^, Supplementary Material, Figure S7)).

### 4.5. Potentiometric Measurements

The constants for protonation, Zn^II^ complexation, and BPA coordination by the Zn^II^ complexes with BPA, were determined by means of potentiometric (pH-metric) titrations in aqueous NaCl 0.1 M at 298 ± 0.1 K. The titrations were performed by using an automated system composed of a 50 cm^3^ reaction vessel, water-thermostatted at 298.1 ± 0.1 K, mounted on a Metrohm 728 stirrer, and equipped with a combined Metrohm 6.0262.100 electrode and a source of nitrogen presaturated with 0.1 M NaCl to maintain an inert atmosphere into the vessel during the measurements. The titrant was delivered by a Metrohm 665 Dosimat buret, while the potentiometric measurements were made with a Metrohm 713 pH meter. The acquisition of the electromotive force (e.m.f.) data was performed with the computer program PASAT [45]. The electrode was calibrated as an hydrogen-ion concentration probe by titration of previously standardized amounts of HCl with CO_2_-free NaOH solutions and determining the equivalent point by Gran’s method [46], which gives the standard potential, *E*°, and the ionic product of water (pK_w_ = 13.73 ± 0.01 in our experimental conditions). All the reagent solutions were prepared by using freshly boiled, doubly deionized water, saturated with anhydrous nitrogen prior to use. The NaOH solution was standardized against carbonate free potassium hydrogen phthalate and stored under nitrogen atmosphere.

Measurements were performed by using a total concentration of [Ru(phen)_2_**L**^2+^] of 1 × 10^−3^ mol/dm^3^, in a 2–11 pH. In the analysis of Zn^II^ binding, [Zn^II^]:[Ru(phen)_2_**L**^2+^] molar ratios were varied from 0.4:1 to 1.8:1 to ascertain the stoichiometries of metal complexes formed in solution. In the analysis of BPA binding in the absence or presence of Zn^II^, the concentration of BPA was varied from 1 × 10^−3^ to 10 × 10^−3^ mol/dm^3^ while Zn^II^ concentration, if present, was 1 × 10^−3^ or 2 × 10^−3^ mol/dm^3^. For each system, at least three titration experiments, consisting of ca 100 data points each, were performed. The equilibrium constants were determined from e.m.f. data by employing the computer program HYPERQUAD [47] while the Hyss program [48] was employed to obtain the relative distribution diagrams of the species present in solution.

### 4.6. Photophysical Measurements

Absorption spectra were recorded using a double beam spectrophometer UV/Vis Perkin Elmer Lambda-45 (Perkin-Elmer, Inc.: Waltham, MA, USA) in the 300–800 nm range. The registration of the emission and excitation spectra was performed using a spectrofluorimeter Perkin Elmer LS55 (Perkin-Elmer, Inc.: Waltham, MA, USA). Excited state lifetime measurements were performed using a spectrofluorimeter Edinburgh Analytical Instruments FLS920 (Edinburgh Instruments Ltd.: Livingston, UK), equipped with a time-correlated single-photon counting device. Measurements were performed using a total concentration of [Ru(phen)_2_**L**^2+^] of 2 × 10^−5^ mol/dm^3^. The concentration of BPA was varied between 0 and 2.25 × 10^−4^ mol/dm^3^.

The plot in Figure 6 has been interpolated according to the following equation:

(1) $$\frac{I_{0}}{I}=\frac{{\left[\mathrm{Ru}\right]}_{t}}{\left\{{\left[\mathrm{Ru}\right]}_{t}-{\left[\mathrm{BPA}\right]}_{t}-\frac{1}{K_{a}}+\sqrt{{\left({\left[\mathrm{Ru}\right]}_{t}+\;{\left[\mathrm{BPA}\right]}_{t}+1/K_{a}\right)}^{2}-4{\left[\mathrm{Ru}\right]}_{t}\times {\left[\mathrm{BPA}\right]}_{t}}\right\}/2}$$

where [Ru]_t_ is the initial concentration of [Ru(phen)_2_**L**]^2+^; [BPA]_t_ is the total concentration of BPA; and K_a_ the apparent association constant between [Ru(phen)_2_**L**]^2+^ and BPA. This equation is valid for static quenching with a 1:1 association in the case of the complete quenching of the luminophore. 

Luminescence quantum yield was determined using [Ru(bipy)_3_]^2+^ in aerated aqueous solution (F = 0.028) as reference compound [49].

## Figures and Tables

**Table 1:** Protonation constant of [Ru(phen)_2_**L**]^2+^ in NaCl 0.1 M aqueous solution and 298 K.

Equilibrium	LogK
[Ru(phen)_2_**L**]^2+^ + H^+^ = [Ru(phen)_2_(H**L**)]^3+^	11.02 (3) ^1^
[Ru(phen)_2_(H**L**)]^3+^ + H^+^ = [Ru(phen)_2_(H_2_**L**)]^4+^	10.15 (4)
[Ru(phen)_2_(H_2_**L**)]^4+^ + H^+^ = [Ru(phen)_2_(H_3_**L**)]^5+^	8.53 (5)
[Ru(phen)_2_(H_3_**L**)]^5+^ + H^+^ = [Ru(phen)_2_(H_4_**L**)]^6+^	7.59 (3)
[Ru(phen)_2_(H_4_**L**)]^6+^ + 2H^+^ = [Ru(phen)_2_(H_6_**L**)]^8+^	7.01 (7)
	**pK_a_**
BPA = BPA(H_−1_)^−^ + H^+^	8.4 (1)
BPA(H_−1_)^−^ = BPA(H_−2_)^2−^ + H^+^	10.9 (2)

^1^ Values in parentheses are errors on the last significant figure.

**Table 2:** Formation constants of the Zn^II^ complexes with [Ru(phen)_2_**L**]^2+^ and of their adducts with deprotonated BPA (indicated with BPA(H_−2_)).

Equilibrium	LogK
[Ru(phen)_2_**L**]^2+^ + Zn^2+^ = {Zn[Ru(phen)_2_**L**]}^4+^	14.75 (5) ^1^
{Zn[Ru(phen)_2_**L**]}^4+^ + H^+^ = {Zn[Ru(phen)_2_(H**L**)]}^5+^	8.68 (3)
{Zn[Ru(phen)_2_(H**L**)]}^5+^ + H^+^ = {Zn[Ru(phen)_2_(H_2_**L**)]} ^6+^	7.15 (6)
{Zn[Ru(phen)_2_**L**]}^4+^ + Zn^2+^ = {Zn_2_[Ru(phen)_2_**L**]}^6+^	6.46 (3)
{Zn_2_[Ru(phen)_2_**L**]}^6+^ + 3OH^−^ = {Zn_2_[Ru(phen)_2_**L**(OH)_3_]}^3+^	15.61 (7)
{Zn_2_[Ru(phen)_2_**L**]}^6+^ + BPA^2−^ = {Zn_2_[Ru(phen)_2_**L**]BPA(H_−2_)}^4+^	7.78 (5)
{[Zn_2_[Ru(phen)_2_**L**]BPA}^4+^ + OH^−^ = [Zn_2_[Ru(phen)_2_**L**]BPA(H_−2_)(OH)}^3+^	5.5 (4)
{Zn_2_[Ru(phen)_2_**L**]BPA(OH)}^3+^ + OH^−^ = [Zn_2_[Ru(phen)_2_**L**]BPA(H_−2_)(OH)_2_}^2+^	3.83 (5)

^1^ Values in parentheses are errors on the last significant figure.

## Data Availability

The data supporting the findings of this study are available within the article and its supplementary material.

## Supplementary Materials

The following are available online, Figure S1: ^1^H-NMR spectra of compound **4**, Figure S2: ^13^C-NMR spectra of compound **4**, Figure S3: ^1^H-NMR spectra of compound **L**, Figure S4: ^13^C-NMR spectra of compound **L**, Figure S5: ^1^H-NMR spectra of [Ru(phen)_2_**L**]^2+^, Figure S6: ^13^C-NMR spectra of [Ru(phen)_2_**L**]^2+^, Figure S7: HR-ESI MS spectra of [Ru(phen)_2_**L**]^2+^.
