# Supplementary material for: Exploring the Ability of Luminescent Metal Assemblies to Bind and Sense Anionic or Ionizable Analytes A Ru(phen)2bipy-Based Dizinc Complex for Bisphenol A (BPA) Recognition
Source: Molecules. 2021 Jan 20;26(3):527. doi: 10.3390/molecules26030527 (PMC7864177; doi:10.3390/molecules26030527)
Supplement: Supplementary file 1 [file molecules-26-00527-s001.pdf]

‘Exploring the ability of luminescent metal assemblies to bind and sense anionic or ionizable analytes. A Ru(phen)$_2$bipy-based dizinc complex for bisphenol A (BPA) recognition’

Luca Conti, Liviana Mummolo, Giammarco Maria Romano, Claudia Giorgi, Gina Elena Giacomazzo, Luca Prodi, and Andrea Bencini

Electronic Supplementary Information (ESI)

1. Characterization of metal compounds

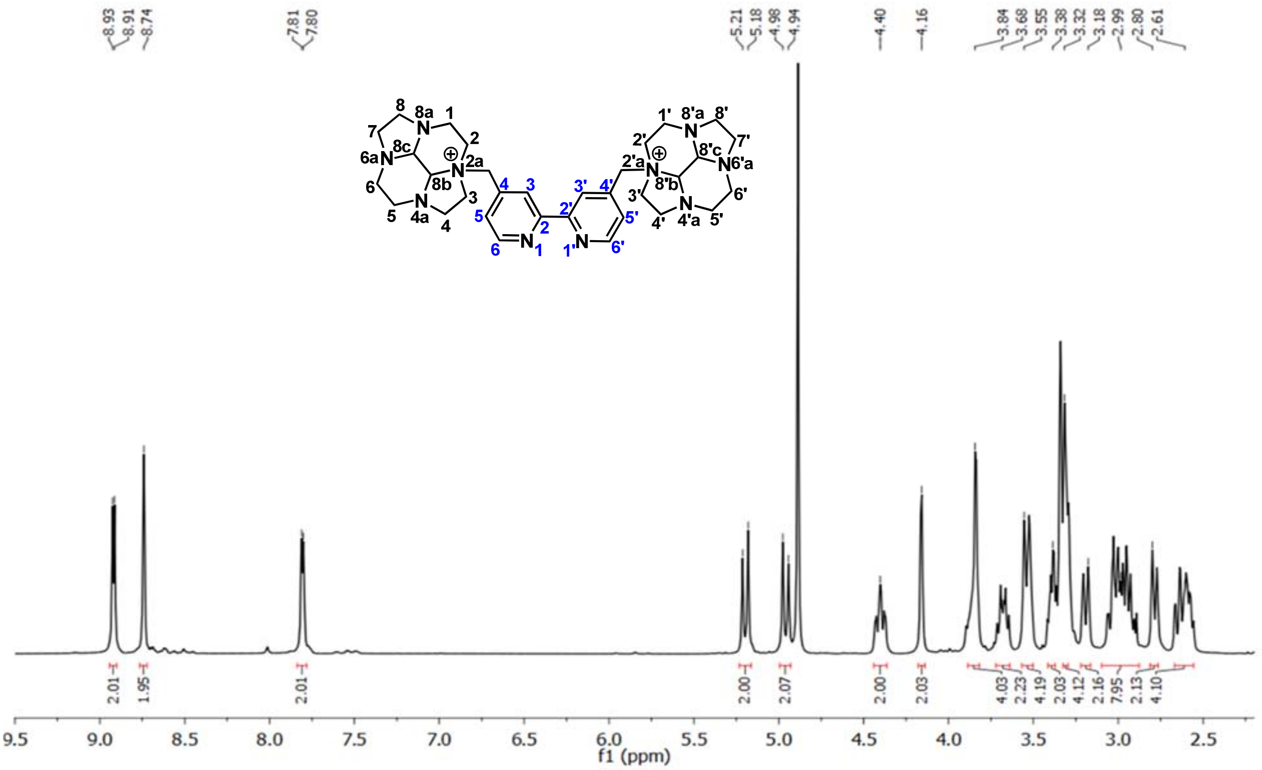

Figure S1. ^{1}H-NMR spectra of compound **4** (400 MHz, CD_3OD). δ(ppm) 8.92 (d, 2H, $J_{6\text{-}5}$ = 4.8 Hz): $H_{6,6'}$ bpy; 8.74 (s, 2H): $H_{3,3'}$ bpy; 7.81 (d, 2H, $J_{5\text{-}6}$ = 4.8 Hz): $H_{5,5'}$ bpy; 5.20 (d, 2H, J=13.2 Hz): $H_{8b\text{-}8'b}$ or $H_{8c\text{-}8'c}$ (glyoxal); 4.96 (d, 2H, J=13.2 Hz); $H_{8b\text{-}8'b}$ or $H_{8c\text{-}8'c}$ (glyoxal); 4.43-4.37 (m, 2H); 4.16 (s, 2H); 3.89-3.84 (m, 4H): -CH_2 (methylen bridge); 3.71-3.65 (m, 2H); 3.55-3.52 (m, 4H); 3.42-3.37 (m, 2H); 3.32-3.29 (m, 4H); 3.21-3.18 (m, 2H); 3.06-2.89 (m, 8H); 2.80-2.77 (m, 2H); 2.66-2.56 (m, 4H).

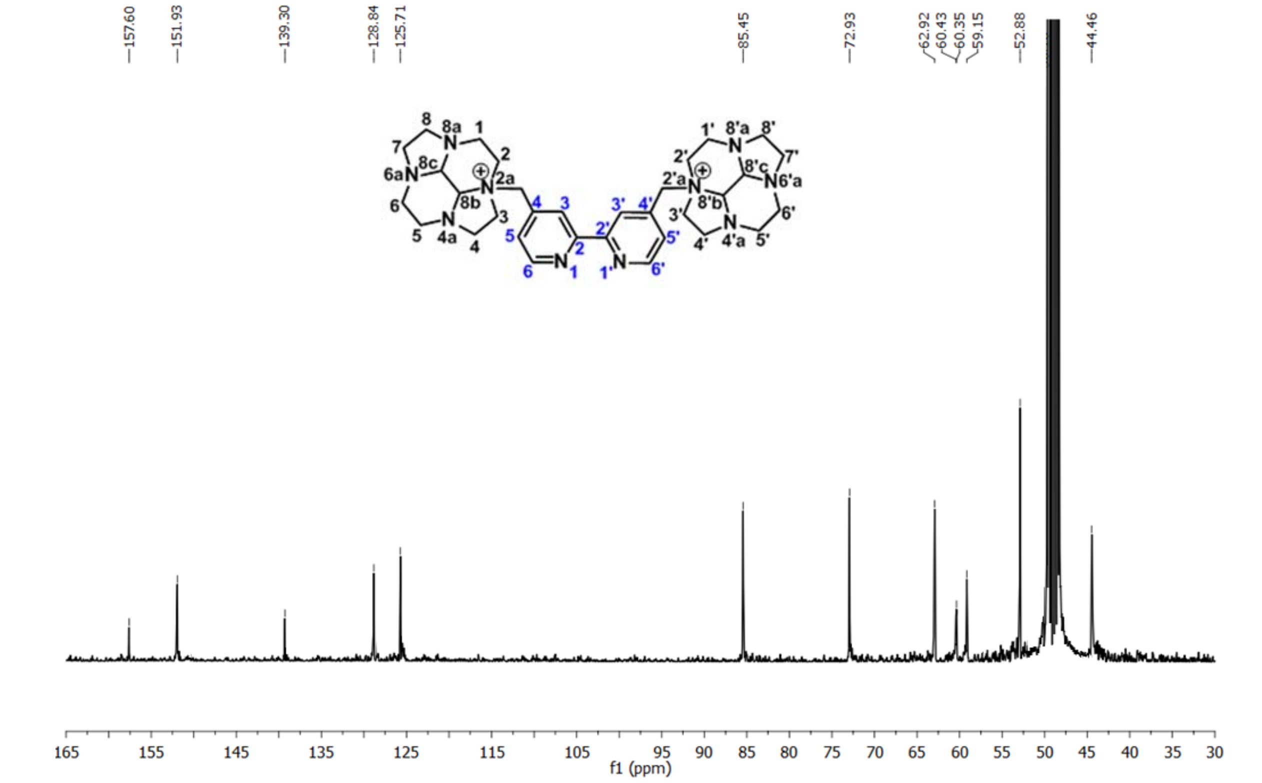

Figure S2. ^{13}C-NMR spectra of compound **4** (400 MHz, CD_3OD). δ(ppm) 157.60: $C_{2,2'}$ or $C_{4,4'}$ (bpy); 151.93: $C_{6,6'}$ (bpy); 139.30: $C_{2,2'}$ or $C_{4,4'}$(bpy); 128.84: $C_{5,5'}$(bpy); 125.71: $C_{3,3'}$(bpy); 85.45: $-CH_2$ (methylen bridge); 72.93; 62.92; 60.43; 60.35; 59.15; 52.88; 44.46.

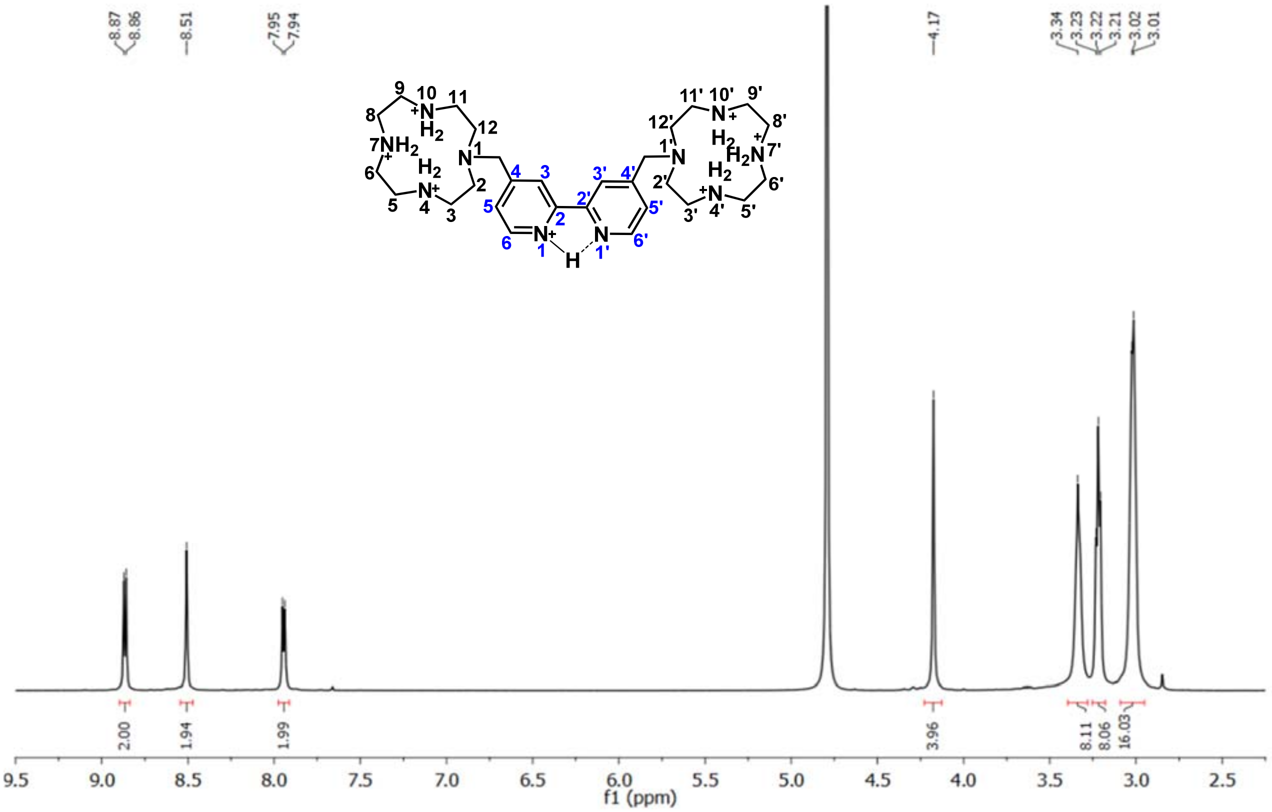

Figure S3. ^{1}H-NMR spectra of compound $H_7\mathbf{L}^{7+}$ (400 MHz, D_2O + DCl, pD < 2). δ(ppm) δ 8.87 (d, 2H, $J_{6\text{-}5}$ = 5.5 Hz): $H_{6,6'}$ (bpy); 8.51 (s, 2H): $H_{3,3'}$ (bpy); 7.94 (d, 2H, $J_{5\text{-}6}$ = 5.4 Hz): $H_{5,5'}$ (bpy); 4.17 (s, 4H): $-CH_2$ (methylen bridge); 3.36-3.32 (m, 8H); 3.24-3.20 (m, 8H); 3.05-2.99 (m, 16H).

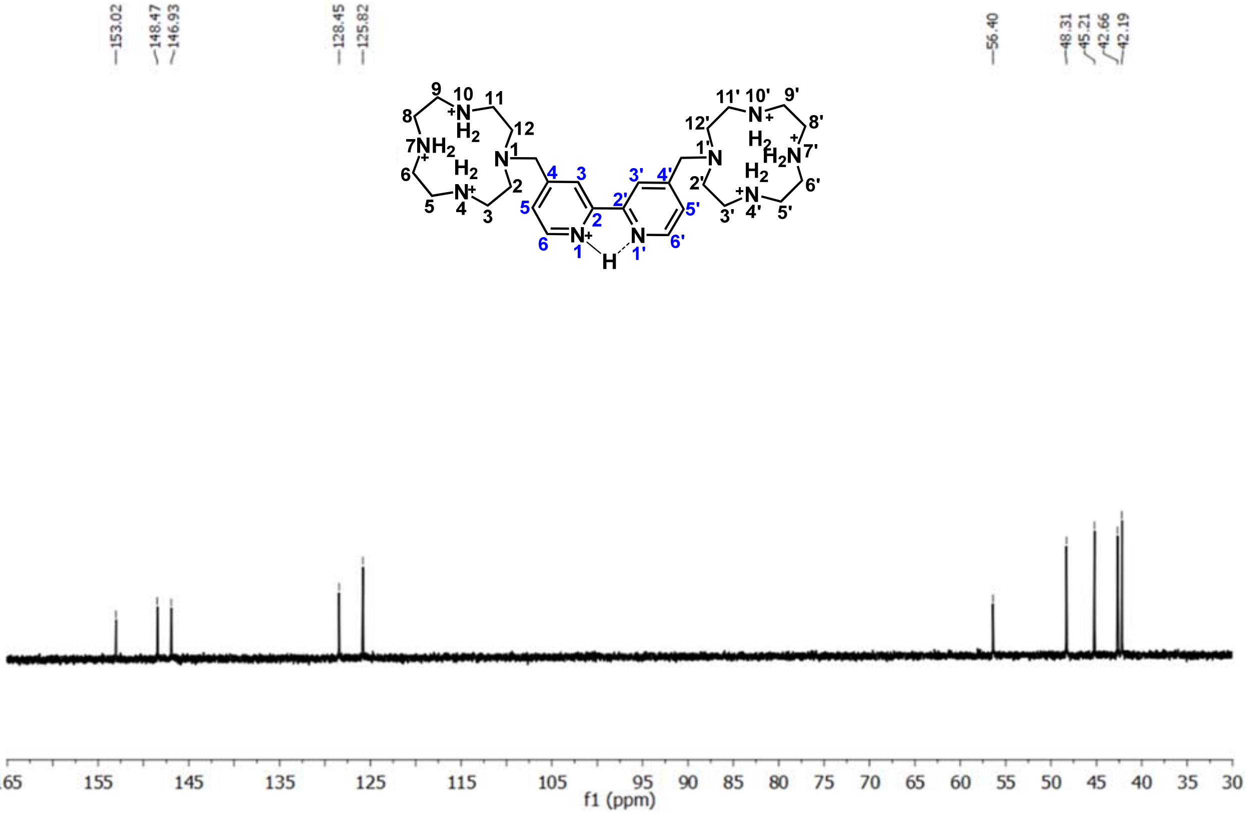

Figure S4. ^{13}C-NMR spectra of compound H_7**L**$^{7+}$ (400 MHz, D_2O + DCl, pD < 2). δ(ppm) 153.02: $C_{2,2'}$ or $C_{4,4'}$ (bpy); 148.47: $C_{2,2'}$ or $C_{4,4'}$ (bpy); 146.93: $C_{6,6'}$ (bpy); 128.45: $C_{5,5'}$ (bpy); 125.82: $C_{3,3'}$ (bpy); 56.40: $-CH_2$ (methylen bridge); 48.31; 45.21; 42.66; 42.19 ppm.

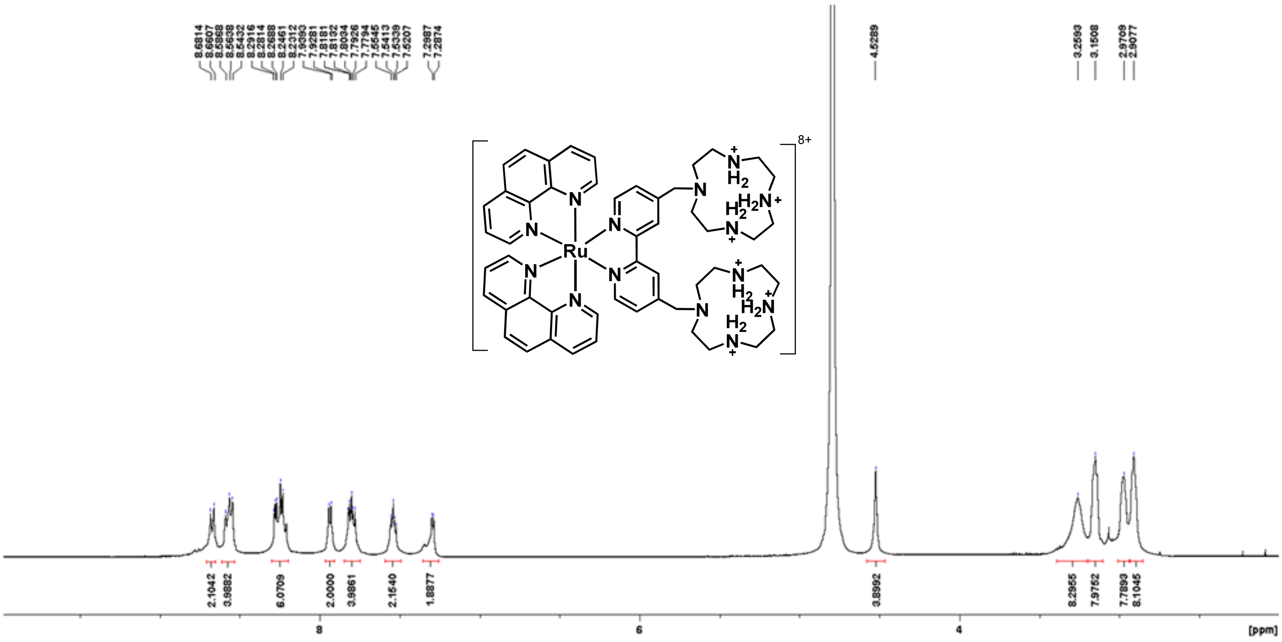

Figure S5. ^{1}H-NMR spectra of compound $Ru(phen)_2$**L**$^{8+}$ (D_2O + DCl, pD < 2, 400 MHz): δ(ppm) 8.67 (d, 2H, J = 8.28 Hz), 8.61-8.53 (m, 4H), 8.33-8.20 (m, 6H), 7.93 (d, 2H, J = 4.48 Hz), 7.84-7.75 (m, 4H), 7.58-7.52 (m, 2H), 7.29 (d, 2H, J = 4.52 Hz), 4.52 (s, 4H, $-CH_2$), 3.37-3.19 (m, 8H), 3.19-3.09 (m, 8H), 3.04-2.94 (m, 8H), 2.94-2.76 (m, 8H).

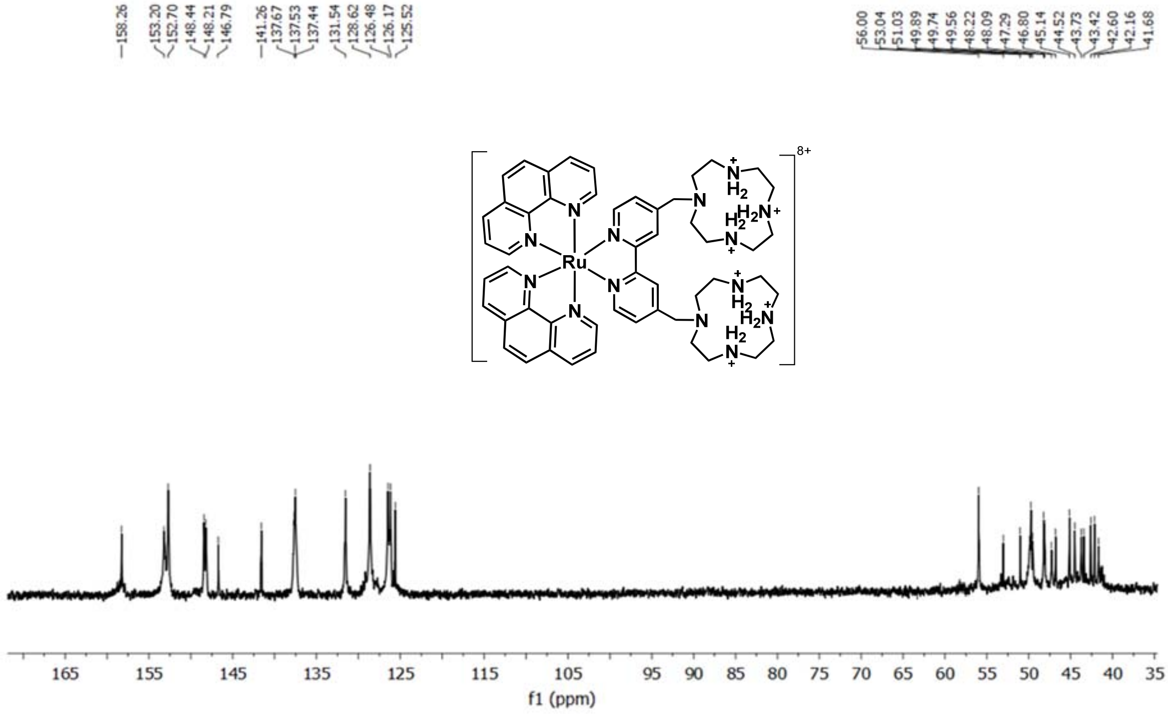

Figure S6. ^{13}C-NMR spectra of compound Ru(phen)$_2$**L**$^{8+}$ (D_2O + DCl, pD < 2, 400 MHz): δ(ppm) 158.26; 153.20; 152.70; 148.44; 148.21; 146.79; 141.26; 137.67; 137.53; 137.44; 131.54; 128.62; 126.46; 126.17; 125.52; 56.00; 53.04; 51.03; 49.89; 49.74; 49.56; 48.22; 48.09; 47.29; 46.80; 45.14; 44.52; 43.73; 43.42; 42.60; 42.16; 41.68.

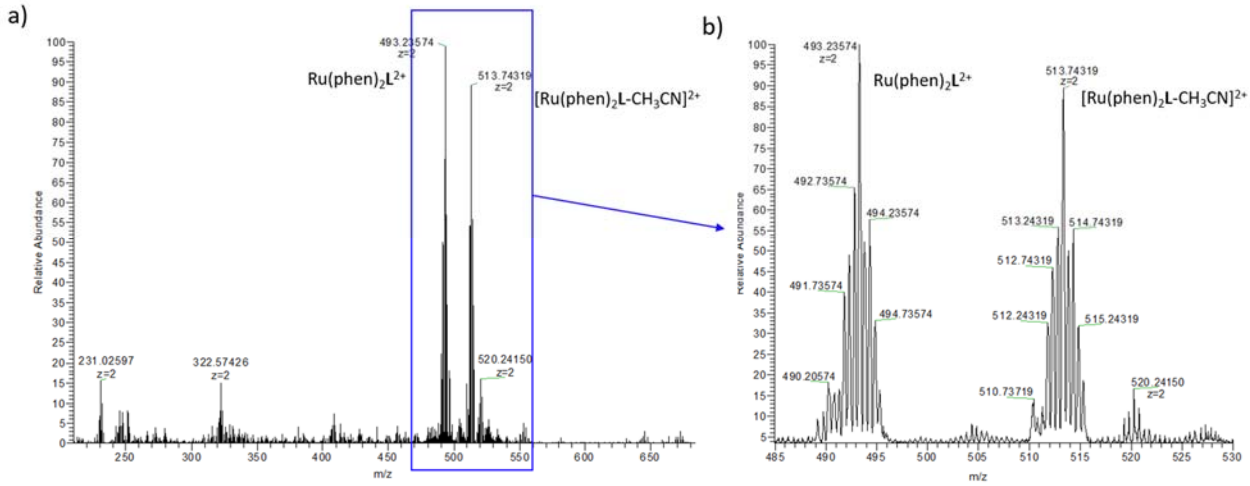

Figure S7. a) HR-ESI MS spectra of compound Ru(phen)$_2$**L**$^{2+}$ in CH_3CN and b) expanded plot showing details of signals of Ru(phen)$_2$**L**$^{2+}$ and of [Ru(phen)$_2$**L**-CH_3CN]$^{2+}$. ESI MS spectrum of Ru(phen)$_2$**L**$^{2+}$ (z = 2): 493.236 (100%); 492.736 (65%); 494.236 (57%); 493.75 (53%); 491.736 (38%); 494.736 (33.3%); 492.236 (48%). ESI MS spectrum of [Ru(phen)$_2$**L**-CH_3CN]$^{2+}$ (z = 2): 513.743 (90%); 513.243 (54.8%); 514.423 (49.2%); 514.743 (53%); 512.743 (45%); 512.243 (31.5%); 515.243 (31%).

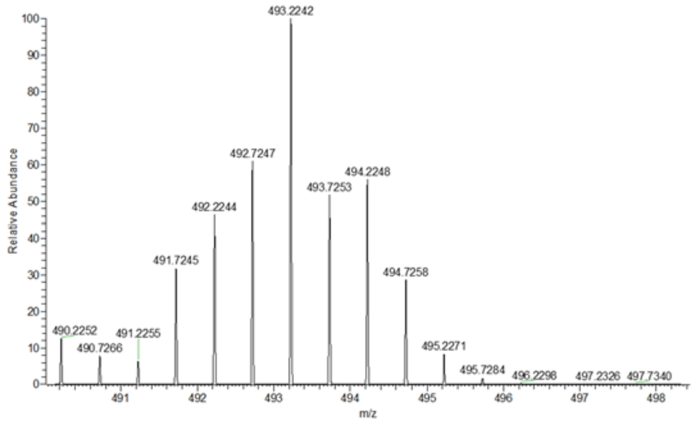

Figure S8. ESI MS set of signals predicted for Ru(phen)$_2$**L**$^{2+}$ (z = 2): 493.22 (100%); 492.73 (61%); 494.22 (56%); 493.73 (52%); 491.72 (32%); 494.72 (29%); 492.22 (46%).

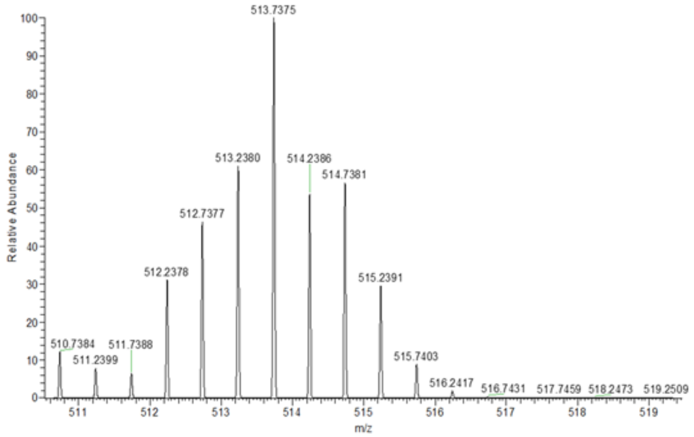

Figure S9. ESI MS set of signals predicted for [Ru(phen)$_2$**L**-CH_3CN]$^{2+}$ (z = 2): 513.74 (100%); 513.24 (61%); 514.24 (52.5%); 514.74 (56.3%); 512.74 (46.2%); 512.24 (31.7%); 515.243 (30%).

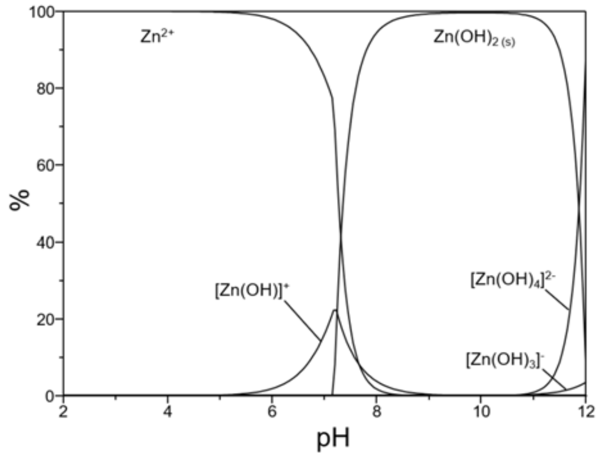

Figure S10. Percentages of the Zn^{II} species formed in water (total Zn^{II} concentration = 1 x 10^{-3} M), including solid $Zn(OH)_2$. The values of the formation constants of the hydroxospecies and the Kps are taken from Gubeli, A. O; Ste-Marie, J. Stabilité des complexes hydroxo et produits de solubilitédes hydroxydes de métaux. I. Argent et zinc. *Can. J. Chem.* **167**, *45*, 826-832

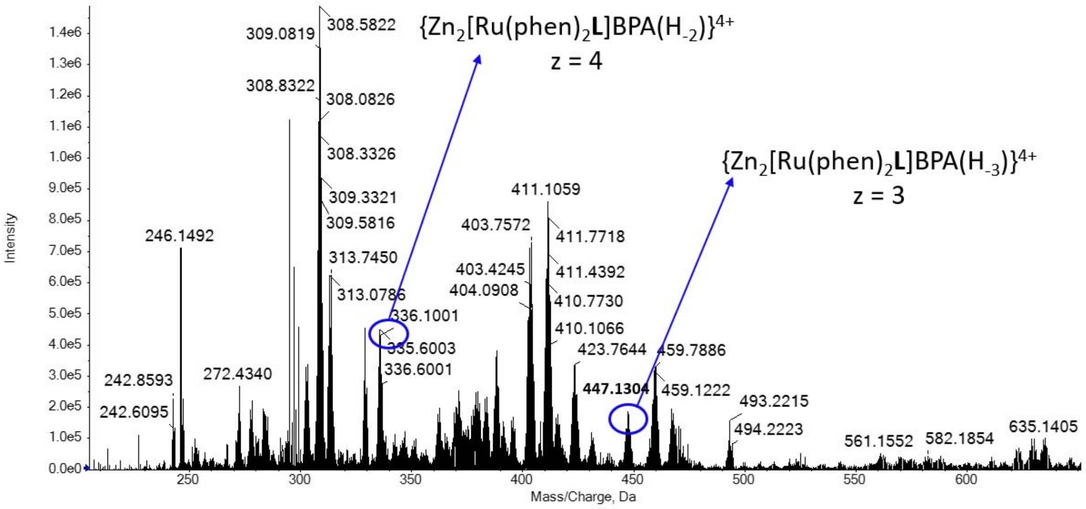

Figure S11. HR-ESI MS spectrum of compound $\{Zn_2[Ru(phen)_2\mathbf{L}]\}^{6+}$ at pH 7 in water in the presence of 1 eq. BPA .

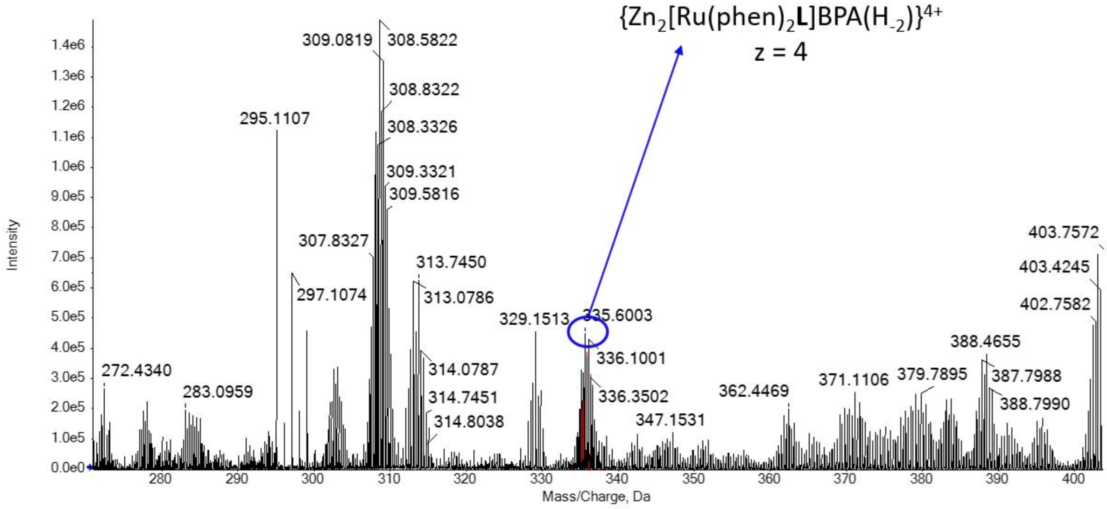

Figure S12. Magnification of the HR-ESI MS spectrum of compound $\{Zn_2[Ru(phen)_2\mathbf{L}]\}^{6+}$ at pH 7 in water in the presence of 1 eq. BPA (260-410 Da region) .

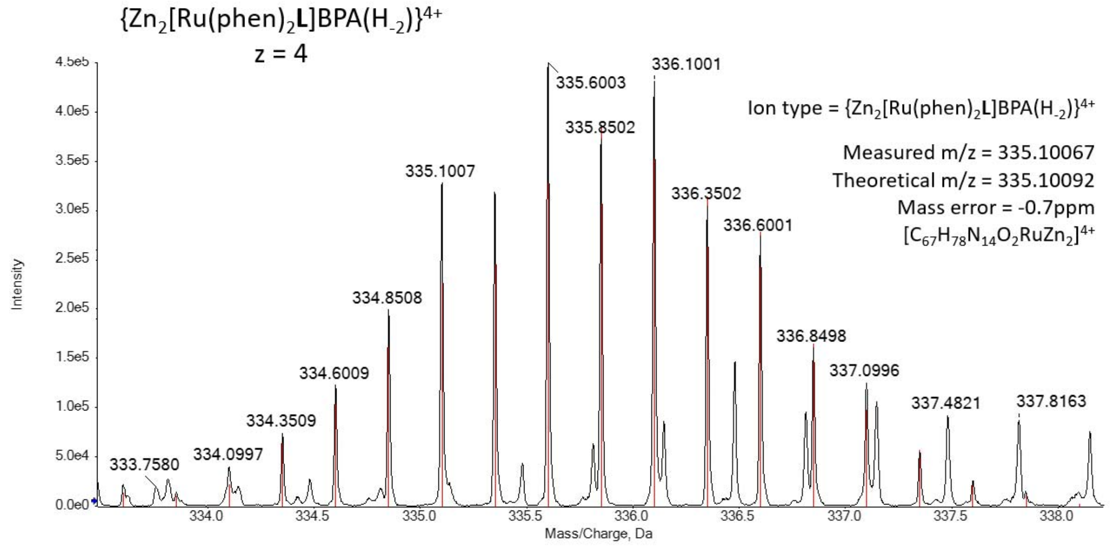

Figure S13. ESI MS set of peaks predicted (red) and observed (black) for compound {Zn_2[Ru(phen)$_2$**L**]}$^{6+}$ at pH 7 in water in the presence of 1 eq. BPA (333.5-338.5 Da region) .

.

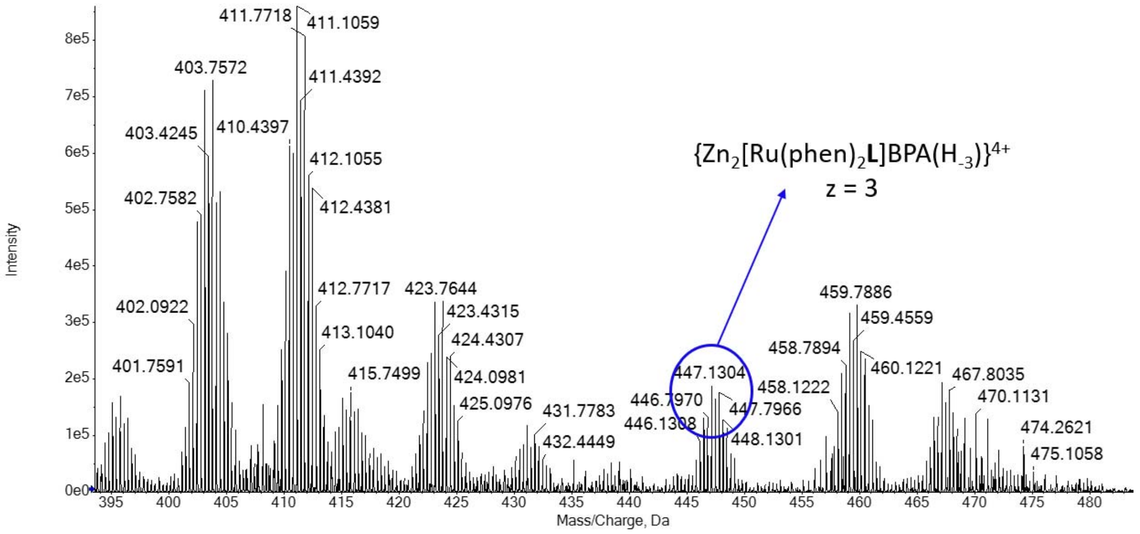

Figure S14. Magnification of the HR-ESI MS spectra of compound {Zn_2[Ru(phen)$_2$**L**]}$^{6+}$ at pH 7 in water in the presence of 1 eq. BPA (390-485 Da region).

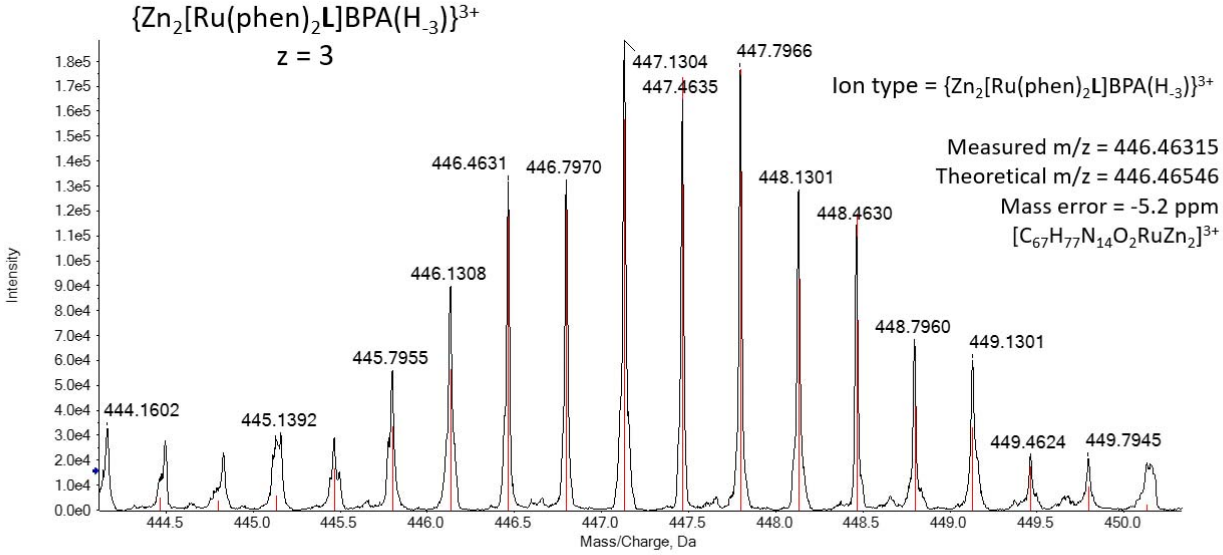

Figure S15. Magnification of the HR-ESI MS spectrum (black: observed peaks; red: calculated peaks) of compound $\{Zn_2[Ru(phen)_2\mathbf{L}]\}^{6+}$ at pH 7 in water in the presence of 1 eq. BPA (444-450.5 Da region).
